# Data-independent acquisition-based quantitative proteomics in plants

**DOI:** 10.3389/fpls.2026.1887480

**Published:** 2026-07-23

**Authors:** Xin Chen, Yu Chen, Wen-Yao Zhang, Mo-Xian Chen, Patrick Willems, Fu-Yuan Zhu

**Affiliations:** 1 State Key Laboratory for Development and Utilization of Forest Food Resources, College of Life Sciences, Nanjing Forestry University, Nanjing, China; 2 Department of Plant Biotechnology and Bioinformatics, Ghent University, Ghent, Belgium

**Keywords:** data-independent acquisition (DIA), high-throughput screening, plants, quantitative proteomics, SWATH-MS

## Abstract

Data-independent acquisition (DIA)-based proteomics is increasingly becoming a standard procedure for comprehensive protein analysis in plant systems. This high-throughput approach is now extensively employed to characterize protein dynamics across various plant physiological processes including growth, development, and stress responses. Particularly in plant science, this approach facilitates the discovery of novel disease resistance proteins and regulatory pathways involved in plant defense mechanisms. Continuing advancements in DIA proteomics methodologies bolster its sensitivity, reproducibility, and throughput, thus broadening its utility in botanical research. This review offers a comprehensive overview of the myriad applications of DIA proteomics in elucidating biological processes and molecular mechanisms with a primary focus on plants. Ultimately, it underscores the pivotal role of DIA proteomics in advancing our understanding of plant biological systems, while also discussing future directions and challenges in the field.

## Introduction

Liquid chromatography coupled with tandem mass spectrometry (LC-MS/MS) serves as the main technology for high-throughput protein identification and quantification (Aebersold and Mann, 2016). Recent advancements in throughput and sensitivity have extended its scope from cataloguing proteins in single or a handful of samples to robust quantification in large-scale projects encompassing hundreds, or even more than thousands of samples (Messner et al., 2023, Messner et al., 2020, Zheng et al., 2025). The ability for extending the number samples have empowered the experimental design and statistical power in a multitude of research fields including personalized medicine, biomarker research, drug screening, plant molecular biology, and genetic association studies. To achieve this goal, the generated large data matrices must prioritize reproducibility, comprehensiveness, and accuracy.

Quantitative proteomics emphasizes precise and reliable measurement of protein abundance across organisms, cells, tissues, time points, and conditions (Noor et al., 2021). It can be categorized into two primary approaches: label-free quantification and labeled quantification. In labeled approaches, peptides are chemically modified with mass tags, and this modification enables downstream multiplexed quantification, which is not applied in label-free quantification. Such chemical mass tags commonly used for multiplexed quantification comprise isobaric reagents such as TMTpro 18-plex reagents (Li et al., 2021) and isobaric tags for relative and absolute quantitation (iTRAQ) (Ross et al., 2004), which generate quantitative MS2 reporter ions after peptide fragmentation. Conversely, non-isobaric mass tags introduce a mass shift of the peptide precursor ions, which is utilized for quantification at the MS1 level. These tags can be incorporated metabolically via stable isotope-labeled amino acids in cell culture (SILAC) (Ong et al., 2002) or mTRAQ chemical reagents (Kang et al., 2010). Although labeled quantification strategies enable sample multiplexing and thus reduced mass spectrometry (MS) analysis time, the reagents themselves can incur relatively high per-sample costs. In addition, quantification can be affected by co-isolation interferences at the MS2 level (Sonnett et al., 2018), a limitation that has driven the development and continued use of synchronous precursor selection based MS3 methods, which substantially reduce ratio distortion in isobaric tagging experiments (Welle et al., 2016).

Unlike labeled quantification methods that rely on expensive stable isotope labels as internal standards, label-free quantification (LFQ) techniques leverage mass spectrometry data obtained during large-scale protein identification and compare the MS1 signal intensities of corresponding peptide precursors across different samples. LFQ operates under the assumption that the same peptide segment exhibits consistent ionization efficiency under identical experimental conditions, and that its MS1 intensity reflects its relative abundance (Pino et al., 2020b). In addition, as peptides elute at similar time across samples in a single chromatography set-up, MS1 peak areas can be retrieved across runs to achieve higher data completeness using so-called matching-between-runs algorithms (Lim et al., 2019). Various computational strategies exist for this peptide-to-protein roll-up, with methods like MaxLFQ offering accurate normalization and protein-level quantification by considering shared and unique peptides across samples. Label-free quantitation is cost-effective because it eliminates the need for expensive isotopic labelling. It also simplifies sample preparation by eliminating labelling steps, thereby reducing potential sources of technical variation and improving reproducibility across large sample sets. Last, it is broadly applicable to a wide range of biological systems, especially when metabolic or chemical labelling is impractical due to sample type or experimental design constraints. Furthermore, with the integration of a data-independent acquisition (DIA) strategies, LFQ can support the simultaneous analysis of over ten samples with a single acquisition method. This multiplexing capability reduces the total number of MS runs needed, thereby saving both time and instrument cost without compromising data completeness.

## Principles and classification of data-independent acquisition

In contemporary proteomics, data acquisition methods can be categorized into two main approaches: data-dependent acquisition (DDA) and DIA. In DDA, the mass spectrometer employs an alternating strategy, cycling between performing a survey scan (MS1) spectrum and a series of data-dependent fragment ion scans (MS2). This acquisition process is inherently constrained by factors such as reproducibility (Geromanos et al., 2009; Tabb et al., 2010), sensitivity, and the speed at which the mass spectrometer can sequentially acquire MS2 spectra (Michalski et al., 2011; Scheltema et al., 2014).

In contrast to DDA approaches, DIA techniques fragment and record MS2 spectra of all precursor ions within predefined isolation windows, allowing for comprehensive MS/MS data across the entire mass range without prior knowledge of specific precursors (Ludwig et al., 2018). As such, DIA mass spectrometry (DIA-MS) methods do not perform peptide precursor selection, effectively circumventing the limitations stemming from inherent irreproducibility and under-sampling associated with DDA.

DIA analysis gained increasing recognition since the sequential window acquisition of all theoretical fragment ion spectra mass spectrometry (SWATH MS) technique by Gillet et al (Gillet et al., 2012), comprising non-overlapping 25 *m/z* windows (**Table 1**). Currently, at least 20 DIA methods have been developed (see **Table 1**). These methods are primarily dependent on the number and size of isolation windows and tailored towards specific instruments. For instance, the ion mobility information captured by timsTOF instruments lead to several optimized acquisition strategies (Meier et al., 2018a; Skowronek et al., 2023). Moreover, the over 200-Hz MS2 scanning speed offered by the recent Orbitrap Astral device (Stewart et al., 2023) has been implemented in a 2 *m/z* isolation narrow-window DIA method (nDIA) (Guzman et al., 2024), thus equaling a typical selected peptide precursor isolation window in DDA.

**Table 1:** Described DIA methods with their associated information.

Method	Precursor *m/z* range	*m/z* window width	Instrument	Data analysis	Reference	Comments
Shotgun CID	Full	Full	QTOF	SEQUEST	(Purvine et al., 2003)	Less controlled fragmentation
Original DIA	400-1400	10	LTQ Orbitrap	SEQUEST	(Venable et al., 2004)	Limited-resolution MS/MS spectra, very long cycle time
MS^E^	300-2000	Full	QTOF	Synapter	(Silva et al., 2005)	Short cycle time, wider precursor m/z range; Increased likelihood of interferences.
PAcIFIC	400-1400	2.5	LTQ Orbitrap	SEQUEST	(Panchaud et al., 2009)	Isolation windows comparable to DDA. Many runs per sample required
AIF	–	Full	Exactive Orbitrap	MaxQuant	(Geiger et al., 2010)	Short cycle time, higher resolution compared to MS^E^; Increased likelihood of interferences
XDIA	–	20	Ion Tap‐ETD‐CAD	XDIA processor	(Carvalho et al., 2010)	High-resolution precursor ion data. Limited-resolution MS/MS spectra
SWATH-MS	400-1200	25	TripleTOF	OpenSWATH	(Gillet et al., 2012)	Optimized and ramped CE per isolation window. Longer cycle time compared to MS^E^
FT‐ARM	500-1000	100	LTQ-FT or LTQ-Orbitrap	–	(Weisbrod et al., 2012)	High-resolution MS/MS spectra. Longer cycle time, reduced precursor m/z range
MSX	500-900	4	Q‐Exactive Orbitrap	Skyline	(Egertson et al., 2013)	Precursor m/z tolerance reducible to 4u. Nonconstant sampling frequency per window, reduced precursor m/z range
WiSIM	400-1000	200/12	Orbitrap Fusion	Pinpoint	(Koopmans et al., 2018)	The range of parent ions was divided into three intervals, and the primary and secondary spectra of each interval were collected independently
Variable window-DIA	400-1200	–	TripleTOF	–	(Zhang et al., 2015)	The parent ion isolation window range was dynamically divided according to the parent ion distribution (PIP) and the total ion current intensity (TIC)
MdFDIA	400-730	10	Orbitrap Fusion Lumos	EASY-nLC system	(Di et al., 2017)	Mass-defect-based multiplex DIA quantification strategy
SONAR	400-900	24	Xevo G2‐XS QTOF	Skyline	(Moseley et al., 2018)	Use wide continuously sliding precursor windows for fragmentation, strengthening the correlation of precursor and fragment ions
BoxCarDIA	400-1200	–	Q Exactive HF Orbitrap	SpectronauntMaxQuant	(Meier et al., 2018a)	The range of parent ion mass charge ratio is divided into several small Windows with independent allocation of maximum ion implantation time to collect parent ion signal
diaPASEF	400-1200	25	timsTOF Pro	OpenSWATH	(Gupta, 2026; Meier et al., 2018b)	Ion mobility separation and quadrupole mass synchronous selection, adding ion mobility dimension information
Scanning SWATH	450-850	5	TripleTOF	DIA NN	(Messner et al., 2021)	The continuous scanning function of the four-stage bar was used to continuously collect the secondary spectra, and the fragment ion intensity was accumulated into bin to increase the information of Q1 ion intensity
PulseDIA	400-1200	–	Q Exactive HF‐X Orbitrap/TripleTOF	DIA‐NN	(Cai et al., 2021)	The isolation window was evenly divided into multiple parts by GPF and allocated to each experiment for independent collection
BoxCarmax	357–1197	10	Orbitrap Fusion Lumos	Spectronaut	(Salovska et al., 2021)	Combine the high sensitivity of BoxCar’s parent ions with the high selectivity of MSX’s parent ions
midiaPASEF	300-1165	25	timsTOF Pro 2	DIA-NN	(Distler et al., 2023)	Maximize information content using overlapping, ion mobility dependent quadrupole windows.
Synchro-PASEF	300-1200	Variable	timsTOF Pro 2	DIA-NN/FragPipe	(Skowronek et al., 2023)	Synchronized movement of quadrupole selection window with the ion mobility scanning.	

**Figure 1:**
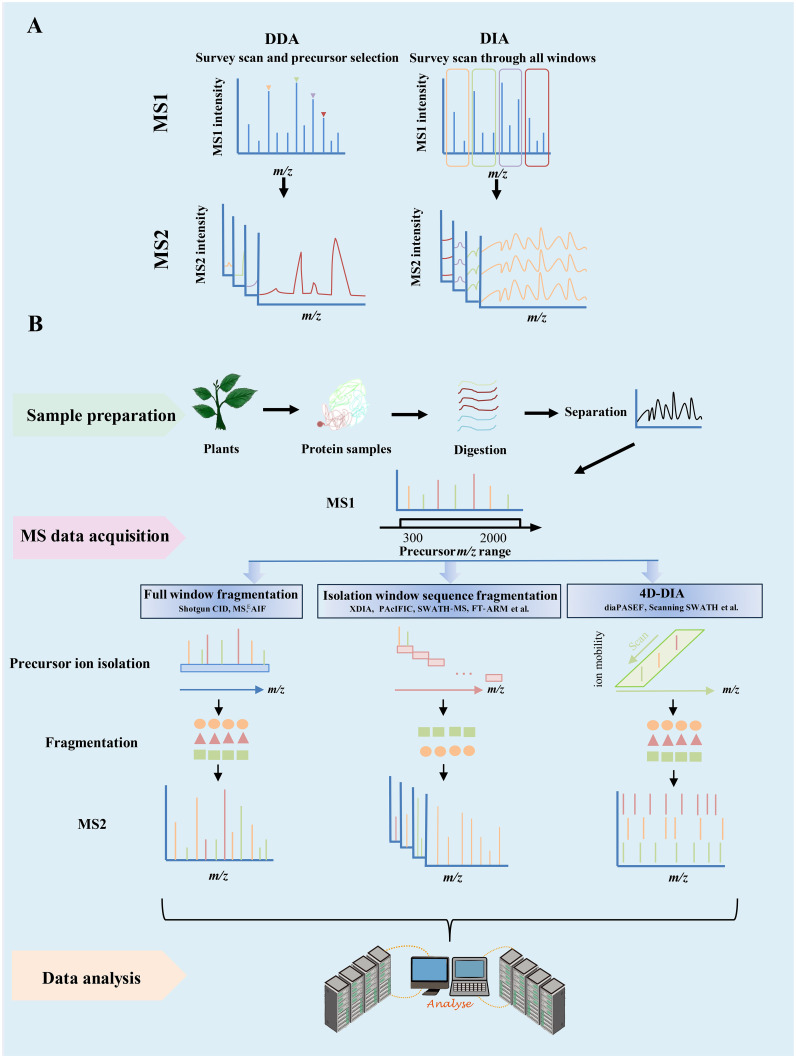
The DIA-MS workflow in plant research. **(A)** The top 20 peptide segments, corresponding to the highest intensity peaks in the primary mass spectrum, were selected for secondary fragmentation in DDA mode, with each secondary spectrum representing a single peptide segment; In DIA mode, the primary mass spectrum is segmented into two stages based on the mass-to-charge ratio, with all relevant data collected. **(B)** The DIA-MS process involves three key stages: sample preparation, data collection, and data analysis. During sample preparation, the target protein is extracted, digested into peptide fragments, and subjected to mass spectrometry sequencing. Various DIA acquisition methods have been developed, which can be broadly classified into three categories based on the number and size of isolation windows and whether additional dimensions are captured for peptide fragments. These include the full-window fragmentation method, the sequential isolation window fragmentation method, and the 4D-DIA method, which incorporates additional data dimensions. The parent ions are then fragmented, and the resulting spectra are collected to obtain information on the fragment ions. Finally, the data are analyzed, and peptide identification is performed through peptide spectrum matching using the mass spectrometry data generated by the DIA method.

Typical sample processing workflows for label-free DIA-MS analysis (**Figure 1**) commonly encompass steps such as protein extraction, digestion, data acquisition, and data processing. However, plant sample preparation remains particularly challenging because rigid cell walls, abundant pigments, phenolic compounds, carbohydrates, and highly abundant proteins often complicate efficient protein extraction and reproducible quantification. To address this bottleneck, an on-filter in-cell digestion strategy has recently been developed to substantially simplify plant sample preparation by bypassing conventional cell lysis, mechanical disruption, detergent-based protein extraction, and extensive clean up steps (Adekanye et al., 2025). Through methanol fixation followed by direct enzymatic digestion within plant tissues, this workflow enables rapid and reproducible processing of challenging plant samples, including leaves and pollen and provides a simple yet powerful strategy for plant proteomics analysis. Next to the acquisition, the data analysis is largely different between DIA and DDA acquired data. Due to the co-fragmentation of all precursor ions, DIA acquisition lacks the direct linkage between precursor and product ions, posing a challenge for traditional (DDA-based) MS search engines. Hence, this necessitates more sophisticated identification algorithms to reconstruct the relationships between product and precursor ions. DIA data analysis algorithms typically employ either spectrum- or peptide-centric approaches (Ting et al., 2015). In the first spectrum-specific method so-called pseudo MS2 spectra are deconvoluted from DIA data, after which traditional search engines can be used to search these generated spectra. Alternatively, spectrum-centric methods employ spectral libraries to retrieve precursor and product ion signals at specified dimensions (such as *m/z*, retention time, possibly ion mobility) and using their expected b/y ion intensities for scoring. Traditionally, such spectral libraries were constructed based by aggregating DDA identification results, which are then transformed into a spectral library where all peptide ions are represented by reference spectra. In contrast, peptide-centric methods directly interrogate DIA data for evidence of specific peptides, using theoretical fragmentation patterns to detect and score ion signals, often without requiring a spectral library (Sultana et al., 2017). Consequently, the efficacy of targeted DIA-MS analysis heavily relies on the composition and depth of the assay library. However, enabled by continuous algorithmic innovations, particularly in the reconstruction of chromatographic elution groups and prediction of fragment ion spectra, there exist DIA-only methods that do not depend on DDA runs for building spectral databases. In such library-free DIA workflows, spectral libraries are generated on the fly by in silico digestion of protein sequences followed by computational estimation of chromatographic properties and fragment ion intensities. This strategy eliminates the dependency on experimental DDA-based libraries and is implemented in tools such as Spectronaut (directDIA), DIA-NN (Demichev et al., 2020) and MaxDIA (Sinitcyn et al., 2021) algorithms (see **Table 2**). These developments, building on foundational work from as early as 2017-2018 (Gessulat et al., 2019), have significantly broadened the applicability and accessibility of DIA-MS across diverse biological contexts (Wen et al., 2022, Wen et al., 2023; Zhong et al., 2020).

**Table 2:** Summary of some DIA algorithms.

Algorithm name	Spectrum/peptide-centric	Predicted spectral library or not	Reference
ProteinPilot	Spectrum-centric	No	(Shilov et al., 2007)
PeakView	Peptide-centric	No	(Gillet et al., 2012)
DeepNovo-DIA	Spectrum-centric	No	(Gillet et al., 2012)
OpenSWATH	Peptide-centric	No	(Röst et al., 2014)
DIA-Umpire	Spectrum-centric	No	(Röst et al., 2014)
Spectronaut	Peptide-centric	Yes	(Bruderer et al., 2015)
Group‐DIA	Spectrum-centric	No	(Li et al., 2015)
PECAN	Peptide-centric	Yes	(Ting et al., 2017)
EncyclopeDIA	Peptide-centric	No	(Searle et al., 2018)
PEAKS	Combine peptide-centric and spectrum-centric	Yes	(Tran et al., 2019)
Skyline	Peptide-centric	No	(Pino et al., 2020a)
CANDIA	Spectrum-centric	No	(Buric et al., 2020)
DeepRT	Spectrum-centric	No	(Ma et al., 2017)
DreamDIA	Peptide-centric	No	(Gao et al., 2021)
DIA-NN	Combine peptide-centric and spectrum-centric	Yes	(Demichev et al., 2020)
DIAmeter	Peptide-centric	No	(Lu et al., 2021)
MaxDIA	Peptide-centric	Yes	(Sinitcyn et al., 2021)
AlphaDIA	Peptide-centric	Yes	(Wallmann et al., 2025)

DIA shares conceptual roots with targeted approaches such as selected reaction monitoring (SRM) and parallel reaction monitoring (PRM), in that both can be interpreted as targeting predefined peptide features across samples (Zhang et al., 2020). Early implementations of DIA, such as the “hyper reaction monitoring” mode in Spectronaut, were explicitly inspired by this targeted framework, treating each peptide as an individual assay, similar to how Skyline processes DIA data (Bruderer et al., 2015). Thus, the distinction between DIA and PRM is more a matter of degree than of kind, and should not be overstated.

In a typical DIA scheme, the instrument isolates and fragments all precursor ions within a defined mass-to-charge (m/z) window. These windows can be wide or narrow, and are tiled sequentially across the full precursor mass range to acquire MS/MS spectra in a cyclical fashion. Compared to full-range MS^all^ fragmentation, which lacks precursor selection altogether, windowed DIA offers improved specificity and better precursor-fragment relationships. Nevertheless, due to the multiplexed nature of DIA data, one of the key analytical challenges is the deconvolution of fragment ions from co-eluting peptides, particularly in complex samples. Early criticisms focused on the loss of direct linkage between precursor and fragment ions. However, various strategies have since been developed to address this limitation. For example, alignment of MS1 and MS2 elution profiles has proven useful for enhancing precursor-fragment pairing (Rosenberger et al., 2017), and some algorithms even incorporate MS1-level quantification such as DIA-Umpire (Tsou et al., 2015). Additionally, recent innovations such as ion mobility separation and sliding quadrupole scanning introduce a fourth dimension, represented by either ion mobility or quadrupole position. These additions extend the conventional 3D-DIA framework (m/z, retention time, and intensity) into a four-dimensional DIA (4D-DIA) approach, significantly enhancing both identification and quantification accuracy (Meier et al., 2020, Meier et al., 2018b; Messner et al., 2021). The incorporation of an additional fourth dimension endows 4D proteomics with several notable advantages compared to traditional methods (Gupta, 2026). First, it enhances peptide separation and identification efficiency; Second, it enables more rapid analysis with increased throughput; Third, it improves the precision of quantitative measurements; Finally, it expands overall proteome coverage, thereby boosting sensitivity and facilitating the detection of low-abundance proteins. Nowadays, an increasing number of researchers are applying 4D-DIA technology to enhance the speed and sensitivity of mass spectrometry scans, achieving a revolutionary improvement in the performance of proteomics in terms of depth of identification, detection cycle, and quantitative accuracy (Barkovits et al., 2018).

## Application of DIA-MS in plant research

**Figure 2:**
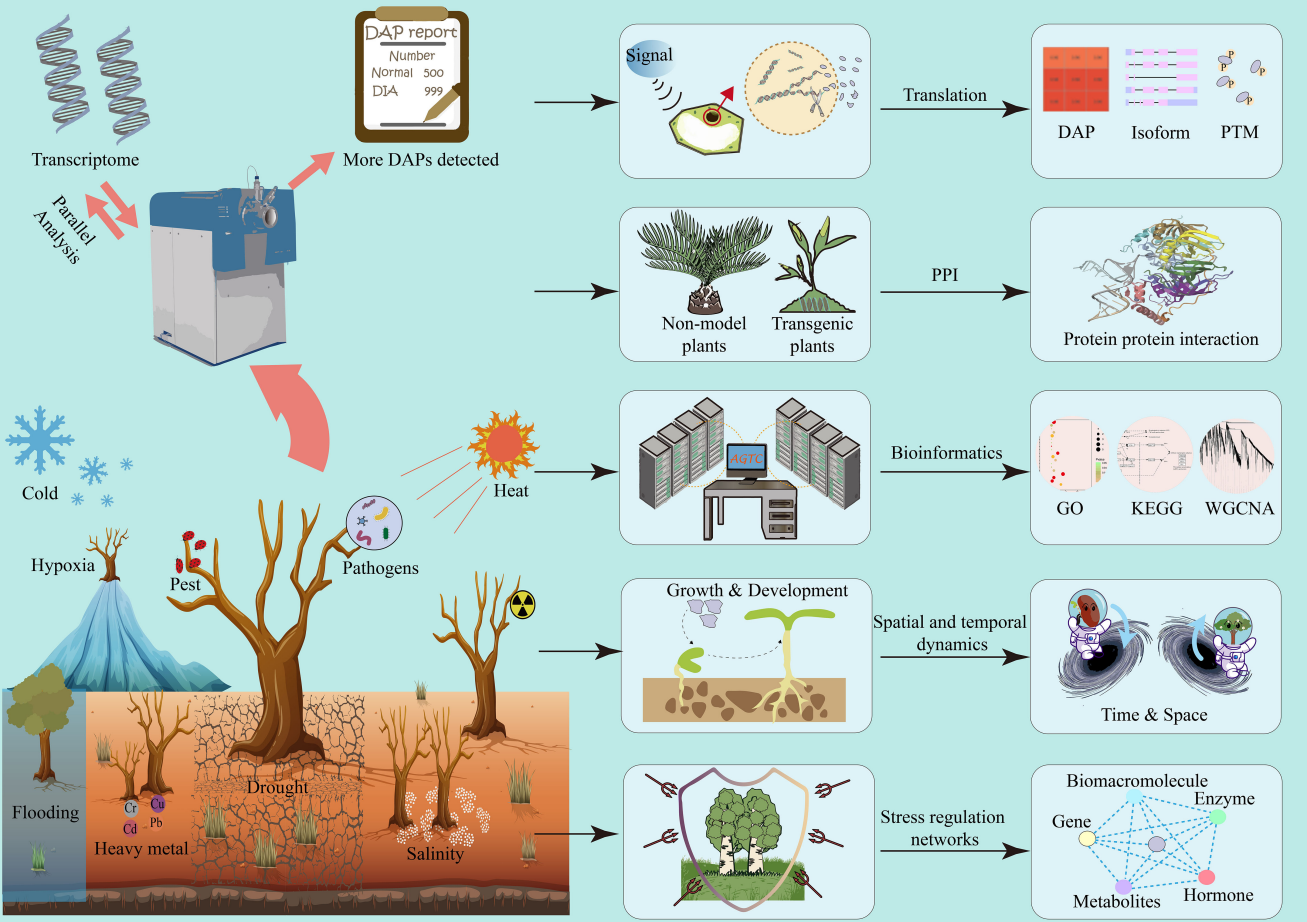
Strategies and applications for DIA-MS in plant research. For plants subjected to biological and abiotic stresses, DAPs were analyzed to study the mechanisms influencing plant response to various stresses, including protein interactions, alternative splicing, PTM, etc., while the transcriptomics and other omics were combined to analyze plant stress regulatory networks. Studies of plant growth and development have also solved the dynamic balance of plant growth in time and space.

While DIA-MS has been extensively employed in animal studies (Ludwig et al., 2018), its application in plant research has progressed relatively more moderately (**Figure 2**). Technological advances in proteomics are typically oriented towards non-plant species such as human cell lines, yeast or *Escherichia coli*. Plant proteomics involves lengthy procedures involving tissue homogenization, cell lysis, and protein extraction require separate optimization for each group due to inherent differences in cell wall structure, secondary metabolites and tissue organization. Nevertheless, there has been a notable increase in research on DIA-MS techniques such as SWATH-MS in plant studies, indicating a growing trend that brings its application closer to that observed in animal research (Gillet et al., 2012). According to recent reviews, proteomics now serves as a definitive bridge between genomic potential and functional phenology, bypassing the limitations of transcript-protein correlation to reveal the spatiotemporal dynamics and post-translational landscapes that govern developmental transitions (Sang et al., 2025). DIA-MS has been conducted in diverse plants, encompassing both model and non-model species such as Arabidopsis (Zhu et al., 2016a), rice (Zhu et al., 2016b), maize (Jia et al., 2018), apple (Chen et al., 2019a), barley (Osama et al., 2021), and others. These studies are largely situated in plant growth and developmental processes as well as environmental stress responses (see **Table 3**).

**Table 3:** Part of fundamental studies on DIA-MS in plant applications.

Species	Topic (stress/tissue)	Type of technology	Findings	Refs
*Arabidopsis thaliana*	Lead stress	SWATH-MS	Proteogenomic analysis showed that ~83.4% of intron-containing genes underwent AS in ABA-treated Arabidopsis seedlings; ABA altered AS patterns and promoted translation of differentially expressed AS isoforms.	(Zhu et al., 2017)
*Persicaria minor*	MeJA	SWATH-MS	SWATH-MS profiled 751 proteins in MeJA-treated *P. minor* leaves and identified 40 significantly changed proteins; MeJA enhanced stress-, lipid metabolism-, secondary metabolism-, DNA/cell wall degradation-related proteins, but suppressed photosynthesis and protein synthesis-related proteins.	(Aizat et al., 2018)
*Arabidopsis thaliana*	Nitrogen	SWATH-MS	SWATH-MS detected 736 nitrogen starvation-responsive proteins in Arabidopsis seedlings, involving amino acid, photosynthesis, lipid and glucosinolate metabolism. GRF6, a nitrogen-induced 14-3–3 protein, was further validated as a positive regulator of nitrogen stress adaptation.	(Zhu et al., 2018)
*Zea mays*	NaCl	SWATH-MS	Under 200 mM NaCl, intact maize seed germination was strongly repressed, while isolated embryos still germinated. RNA-seq and SWATH-MS revealed distinct embryo/endosperm responses, endosperm-dependent ABA accumulation, and AS-mediated early salt-stress regulation.	(Chen et al., 2021)
*Zea mays*	Drought	nano-HPLC-MS/MS	DIA-MS identified 826 drought-responsive DAPs in two maize hybrids at the kernel-filling stage, including 7 DAPs specifically accumulated in drought-tolerant ND476. Chaperones, DNA replication-related enzymes, vicilin, and ABA-responsive RAB17 were linked to starch/sucrose metabolism, terpenoid biosynthesis, cyanoamino acid metabolism, and carbon metabolism.	(Yang et al., 2020)
*Solanum tuberosum*	*Phytophthora infestans*	SWATH-MS	SWATH-MS identified 4,401 proteins and 1,429 PAMP-responsive DEPs in potato after *P. infestans* culture filtrate treatment; six candidates, including MPT3, VAMP714, LysoPL2, APX1, HSP70-2, and FKBP15-1, were validated as PTI-related proteins.	(Mu et al., 2022)
*Oryza sativa*	*Cnaphalocrocis medinalis* insect pest	SWATH-MS	SWATH-MS quantified 1,732 proteins in resistant Qingliu and susceptible TN1 rice during *Cnaphalocrocis medinalis* herbivory. Qingliu showed higher abundance of PAL, CHS, and LOX before and during early herbivory, suggesting primed flavonoid biosynthesis and JA signaling; amino acid biosynthesis and glutathione-mediated antioxidation were activated at 72 h.	(Cheah et al., 2020)
*Arabidopsis thaliana*	Lead	SWATH-MS	SWATH-MS quantified 1,719 proteins and identified 231 Pb-responsive proteins in Arabidopsis, with 151 increased and 80 decreased under Pb stress. JA- and GSL-related pathways were induced, and JA-deficient/insensitive mutants showed Pb hypersensitivity, indicating that JA mediates Pb stress responses.	(Zhu et al., 2016a)
*Glycine max* (L.) Merr.	Soybean rust caused by *Phakopsora pachyrhizi*	diaPASEF	4D-DIA identified 12,852 proteins and 1,510 DEPs during soybean rust infection. Resistant soybean activated CRKs, LRR-RLKs, MAPK signaling, cell wall reinforcement, calcium/ROS pathways, and phenylpropanoid–isoflavonoid metabolism, while susceptible plants suppressed photosynthesis and defense; qPCR confirmed 84% of tested DEPs.	(Lu et al., 2025)
*Zea mays*	Ear, tassel, immature embryo, and root	SWATH-MS	SWATH-MS identified 4,551 proteins across maize ear, tassel, immature embryo, and root; integrated transcriptome–proteome analysis revealed weak-to-moderate mRNA–protein correlations and tissue-specific enrichment of reproductive development, lipid/fatty acid biosynthesis, and redox-related proteins.	(Jia et al., 2018)
*Mikania micrantha*	Stem	SWATH-MS	DIA-MS and RNA-seq identified 5,196 proteins from *M. micrantha* stems and roots; DEGs/DEPs were enriched in photosynthesis/carbon fixation, and PSI/PSII and cytochrome b6/f proteins accumulated in adventitious roots, supporting rapid stem growth and vegetative propagation.	(Cui et al., 2021)
*Oryza sativa*	Late-flowering inferior spikelets (IS); earlier-flowering superior spikelets (SS)	SWATH-MS	SWATH-MS identified 304 DEPs between rice inferior and superior spikelets during grain filling, with 241 decreased and 63 increased proteins in IS. DEPs were mainly involved in photosynthesis, protein metabolism, and energy metabolism, suggesting post-transcriptional regulation of poor grain filling in IS.	(Zhu et al., 2016b)
*Oryza sativa*	Straw carbon reserves	SWATH-MS	SWATH-MS/RNA-seq showed that moderate soil drying promoted rice straw carbon remobilization during grain filling by upregulating starch-degradation proteins, SPS8/SPP1-mediated sucrose synthesis, and sugar transporters including MSTs and SUT2.	(Wang et al., 2020)
*Oryza sativa*	Seed germination	SWATH-MS	AP-SWATH quantified 1,872 rice embryo proteins and identified 288 carbonylated peptides from 144 proteins during germination; 66 dynamically carbonylated proteins were linked to ROS homeostasis, ABA regulation, and seed reserve metabolism, with peroxiredoxin showing 23 carbonylated peptides and increased activity.	(Zhang et al., 2016)
*Hordeum vulgare*	Seed	SWATH-MS	DIA-MS showed that barley germination is marked by rapid loss of desiccation-tolerance and germination-inhibitory proteins, followed by decreased lipid/protein/nutrient-storage proteins. Dormant seeds showed distinct changes in sulfur metabolic enzymes, α-amylase/trypsin inhibitors, and histone proteins.	(Osama et al., 2021)
*Solanum lycopersicum*	Fruit; cuticle	WiSIM-DIA	WiSIM-DIA identified 1,140 tomato fruit proteins and 67 DEPs between cd2 and WT M82, with 53 proteins reduced in cd2. The reduced abundance of CUS1 supported the role of CD2 in cutin biosynthesis, while other DEPs implicated cell wall, anthocyanin, development, and stress-response pathways.	(Martin et al., 2016)
*Malus pumila* Mill	Bud; fruit skin color	SWATH-MS	SWATH-MS identified 451 DEPs between Fuji apple skin and its red-skin bud sport mutant; the mutant accumulated more anthocyanins and upregulated photosynthesis-, stress-, and anthocyanin biosynthesis-related proteins, while MAPK4 and MVK were downregulated.	(Chen et al., 2019a)
*Lycoris*	Regulatory metabolism	SWATH-MS	SWATH-MS quantified 2,193 proteins in three Lycoris species; 720 DEPs in high-alkaloid *L. longituba* and 463 DEPs in L. incarnata were identified relative to low-alkaloid *L. sprengeri*. DEPs were enriched in amino acid metabolism, starch/sucrose metabolism, and galanthamine biosynthesis.	(Tang et al., 2023)
*Osmanthus fragrans*	Androdioecy breeding	SWATH-MS	SWATH-MS identified 2,298 proteins and 428 DEPs between male and hermaphroditic pistils of *O. fragrans*, with 210 upregulated and 218 downregulated in males. DEPs were enriched in carbohydrate metabolism, secondary metabolism, and signaling, suggesting roles of glucose metabolism, flavonoids, isoprenoids, lignins, and post-transcriptional regulation in the androdiecious breeding system.	(Duan et al., 2021)
*Nepenthes × ventrata*	Digestive organ	SWATH-MS	SWATH-MS identified 32 pitcher-fluid proteins in *Nepenthes* × *ventrata*, including 19 quantifiable proteins and 16 previously unreported proteins. The study showed prey-independent protein replenishment after pitcher opening and identified a new secreted aspartic proteinase Nep6.	(Zakaria et al., 2019)

### Studying plant stress adaptation and phytoremediation

DIA-MS offers superior reproducibility and inter-run consistency through systematic, unbiased acquisition of all precursor ions within predefined m/z windows. This acquisition strategy enhances proteome coverage, particularly for low-abundance peptides often missed by DDA. By minimizing redundant sampling of high-abundance species, DIA improves acquisition efficiency. Furthermore, the comprehensive recording of fragment ion spectra ensures high data completeness and allows retrospective analysis using updated spectral libraries, making DIA a robust platform for large-scale plant proteomics (Zhang et al., 2019).

In an early study, SWATH-MS was used to quantify protein changes in response to lead (Pb) toxicity, revealing a role in jasmonic acid (JA) phytohormone signaling (Zhu et al., 2016a). This discovery offered a novel perspective of plant adaptation to heavy metals. Pb tolerance was also studied by DIA-MS in poplar trees, offering molecular targets and strategies for phytoremediation (Zhu et al., 2016b), while DDA might overlook these critical low-abundance regulators due to stochastic sampling. More recently, by joining forces of transcriptomics for in silico protein database generation and DIA-MS, a proteogenomic analysis was conducted to study Pb tolerance in poplar (Zhu et al., 2023). Here, a robust Pb-inducible chaperone protein, PtHSP70, with two splicing isoforms, was identified through the integration of proteomics and transcriptomics. This discovery relied on DIA-MS’s ability to reproducibly quantify splice isoforms across samples, a task challenging for DDA due to inconsistent precursor selection. Additionally, DIA’s retrospective data-mining capability allowed reanalysis of archived datasets for novel Pb-binding proteins.

Another study administered the JA derivative methyl-JA to the non-model, herbal species *Persicaria minor*, which triggered a stress response inducing secondary metabolism and suppressing growth and proteins involved in plant growth and development (Aizat et al., 2018). This library was then leveraged to study the effect of another stress phytohormone abscisic acid (ABA) on rosette leaves, revealing differential abundance of approximately 1,800 proteins (Zhang et al., 2019). Compared with DDA, the DIA-MS workflow ensured higher data completeness and reproducibility, enabling a more confident quantification of stress-responsive proteins across biological replicates.

DIA-MS has also been applied to study the effects of nutrient availability and starvation in plants. For instance, proteome changes has been monitored under nitrogen starvation in Arabidopsis seedlings (Zhu et al., 2018) and early responses to salt stress during maize seed germination using DIA-MS (Chen et al., 2021), the latter pinpointing to alternative splicing as regulatory mechanisms (Do et al., 2025; Liu et al., 2024). Unlike DDA, which may miss transient or low-abundance peptide signals, DIA’s comprehensive acquisition allowed these subtle but functionally significant protein changes to be detected. Other studies using DIA-MS elucidated novel pathways associated with hypoxic germination in rice (Chen et al., 2019b), further demonstrating its capability to capture a broader and more reproducible proteomic landscape under dynamic physiological conditions.

Lastly, DIA-MS has been used to study plant-pathogen interactions and exploring the role of plant disease resistance proteins. For instance, this approach has proven valuable in uncovering complex protein networks involved in the resistance response of potatoes to late blight, thereby enhancing the understanding of PTI mechanisms (Mu et al., 2022). Additionally, a distinct defense response characterized by JA signaling, carbon remobilization, and the production of flavonoids and glutathione was identified in *Cnaphalocrocis medinalis*-resistant rice in another study (Cheah et al., 2020). Moreover, the latest 4D-DIA analysis identified the soybean rust disease caused by *Phakopsora pachyrhizi* in soybeans, providing a protein-level blueprint for soybean rust resistance, and identifying candidate targets for marker-assisted breeding and genetic engineering to develop durable disease-resistant varieties (Lu et al., 2025).

### Plant growth and development

DIA-MS is routinely employed in the study of plant growth and development to monitor protein levels associated with differentiation and function of particular plant organs. DIA-MS offers more comprehensive and reproducible coverage across diverse tissues, reducing the risk of missing low-abundance but biologically relevant proteins. For example, a quantitative proteome analysis encompassing maize ear, tassel, immature embryo, and root was conducted using SWATH-MS, resulting in the identification of numerous DAPs (Jia et al., 2018). This analysis revealed distinct tissue-specific protein expression patterns, such as flower development-related proteins in tassel and ear tissues, lipid metabolism proteins in immature embryos, and redox homeostasis proteins in roots. Such patterns might be partially overlooked in DDA workflows owing to the stochastic nature of precursor selection. In rice, DIA-MS has been employed to compare inferior spikelets (IS) with earlier-flowering, superior spikelets (SS) to elucidate protein dynamics influencing grain filling (Zhu et al., 2016b). This revealed a reduced abundance of photosynthetic proteins as well as protein translation and degradation complexes in IS, highlighting the metabolic shifts associated with carbon reserve mobilization from rice straws. Accordingly, under moderate soil drought, DIA-MS further detected upregulation of proteins facilitating such mobilization (Wang et al., 2020), illustrating DIA-MS’s ability to capture stress-induced proteomic changes with high reproducibility across biological replicates. Next to maize and rice, the major food crop barley has also been subjected to DIA-MS analysis to track protein changes throughout seed germination. This revealed imbibition to decrease the abundance of germination inhibitors and desiccation tolerance proteins, after which in a next phase storage proteins showed reduced abundance, thus highlighting key processes during seed germination that serve as putative targets for selective breeding (Osama et al., 2021). The consistent quantification of these temporal protein changes underscores DIA-MS’s advantage over DDA in time-course experiments, where complete datasets are crucial for accurate biological interpretation.

Some studies directly investigate the developmental role of master regulators using DIA-MS. For instance, wild-type and tomato (*Solanum lycopersicum*) plant mutants lacking the CUTIN DEFICIENT2 (CD2) transcription factor, playing a role in tomato cuticle formation, were subjected to DIA-MS, revealing among others CD2 as a driver of cuticle formation (Martin et al., 2016). DIA-MS has also been applied to non-model plant species of interest due their secondary metabolism. Three *Lycoris* spp. Amaryllidaceae plant species, typically used in traditional Chinese medicine, that are rich in alkaloids were investigated by DIA-MS (Tang et al., 2023). Between the three species significant proteome changes in central metabolism and galanthamine biosynthesis was apparent, highlighting the regulatory metabolism of Amaryllidaceae alkaloids in *Lycoris*. Likewise, sweet osmanthus (*Osmanthus fragrans)* was profiled using DIA-MS to investigate sex-associated proteomic differences (Duan et al., 2021). Such findings benefit from DIA-MS’s unbiased and library-driven acquisition, which is particularly advantageous when studying non-model organisms lacking complete reference proteomes. Next to reproductive processes, DIA-MS has been implemented to study protein levels associated with certain phenotypical traits. For example, a DIA-MS study focusing on Fuji apple skin color highlights a clade of regulatory proteins potentially of interest for molecular breeding of apple skin color (Chen et al., 2019a).

### Single-cell plant proteomics

Single cell proteomics (SCP) analysis helps elucidate the molecular basis of different cell types and heterogeneity. DIA-MS has been applied to the quantitative analysis of proteins in SCP, enabling deep proteome coverage (Brunner et al., 2022). Given the extremely limited sample amounts and the critical importance of data completeness in this field, the comprehensive and reproducible acquisition strategy of DIA makes it particularly well-suited for low-input proteomic analyses, such as those involving single cells. Recently, Gebreyesus et al. combined microfluidic chips for all-in-one proteomic sample preparation and DIA-MS for proteomic analysis down to the single-cell level (Gebreyesus et al., 2022), the integrated SciProChip-DIA workflow demonstrated sensitive proteome coverage of 1500 ± 131 protein groups from a single PC-9 cell. This demonstrates DIA’s sensitivity and scalability when integrated with microscale workflows. To further enhance throughput, Derks et al. introduced plexDIA, which applies isobaric labelling (mTRAQ 3-plex) to enable multiplexed single-cell quantification (Derks et al., 2023). This approach achieved quantification of ~1,000 proteins per cell with 98% data completeness, showcasing the advantages of DIA in high-throughput settings. More recently, the application of advanced mass spectrometry platforms such as timsTOF Ultra and Orbitrap Astral, in combination with library-free DIA-MS workflows, has pushed the depth of single-cell proteome coverage to thousands of proteins (2,000 up to 4,000 proteins) per cell (Ctortecka et al., 2024), demonstrating the scalability and robustness of DIA in cutting-edge proteomic research.

To date, most single-cell research in botany has been confined to RNA-sequencing approaches, which yield only indirect readouts of functional cellular activities. Applying SCP to botanical systems introduces unique hurdles, such as sample isolation and interfering macromolecules. Although fluorescence-activated cell sorting (FACS) is a standard method for isolating single cells across biological kingdoms, plant tissues present distinct obstacles. The rigid cell wall encapsulating plant cells must be enzymatically removed before the resulting protoplasts can be partitioned via FACS or alternative sorting technologies (Clark et al., 2022). Specifically, Montes et al. (2024) demonstrated the feasibility of SCP in roots by utilizing fluorescence-activated cell sorting (FACS) to examine 756 individual cells from the *Arabidopsis thaliana* cortex and endodermis. This differentiation successfully facilitates the identification of biomarkers and candidate proteins essential for functional genomics (Montes et al., 2024). Expanding this methodology to aboveground tissues, Fulcher et al. (2026) introduced an optimized, DDA-based plant SCP workflow that integrates tape-sandwich protoplasting and sample preparation with advanced mass spectrometry, achieving high-precision quantification of over 3,000 proteins from a single Arabidopsis leaf mesophyll cell (Fulcher et al., 2026).

Despite these foundational DDA-based breakthroughs in both root differentiation and leaf stress profiling, the implementation of DIA-MS in plant SCP needs further investigation. Transitioning to advanced DIA workflows holds immense potential to unlock a deeper layer of the botanical proteome; its superior data completeness and strict reproducibility can significantly minimize the missing value problem inherent in low-input DDA scans, thereby enabling a more comprehensive characterization of low-abundance regulatory proteins across diverse plant cell types (Bubis et al., 2025).

### Post-translational modifications

DIA-MS has also been used for profiling specific post-translational modifications (PTMs). As most studied PTM, phosphorylation has been quantified by DIA-MS by relying on experimental DDA-based spectral libraries (Bekker-Jensen et al., 2020; Skowronek et al., 2022). Notably, compared with DDA, the DIA approach yielded nearly twice as many identified phosphopeptides (close to 28,000 phosphorylated peptides) and achieved superior reproducibility (R² = 0.93 vs. 0.89), with a dynamic range approximately one order of magnitude higher, underscoring its advantage in detecting low-abundance phosphorylation events and ensuring data completeness in large-scale phosphoproteomic studies. Notably, DIA methods have been shown to identify a substantially higher number of phosphopeptides and phosphorylation sites compared with DDA, with improved reproducibility, thereby enabling more robust and comprehensive phosphoproteomic profiling in large-scale studies. Alternatively, a library-free approach was demonstrated in Spectronaut using a spectrum-centric approach where generated pseudospectra can be searched for phosphorylation (Bekker-Jensen et al., 2020). In addition, predictive models have been trained to identify phosphorylated and ubiquitinated peptides (Zeng et al., 2022), facilitating the generation of forecasted spectral libraries for DIA phosphoproteomics (Lou et al., 2021), further expanding DIA’s applicability to diverse PTMs. Furthermore, a hybrid-DIA strategy integrating targeted and discovery-based acquisition has been developed to achieve sensitive and accurate phosphosite quantification while simultaneously enabling global phosphoproteome profiling, even from limited sample amounts (Martínez-Val et al., 2023). Building on these methodological advances, DIA-based phosphoproteomics has been successfully applied to decode rapid and early signaling networks in plants under various environmental stimuli. For instance, it was utilized to investigate early temperature stress responses, revealing dynamic protein phosphorylation and dephosphorylation events in Arabidopsis seedlings within minutes of cold exposure, which underscored the high sensitivity of DIA-MS in capturing transient signaling cascades (Tan et al., 2021). Similarly, in horticultural crops, DIA-based phosphoproteomic profiling in tomato successfully mapped rapid cold-responsive phosphorylation events, providing crucial insights into the postharvest chilling injury mechanisms at the post-translational level (Tan et al., 2025). Beyond thermal stress, DIA-based phosphoproteomics has also been instrumental in dissecting osmotic and calcium signaling, leading to the identification of early, specific phosphorylation events in Arabidopsis in response to mannitol and EGTA treatments (Sang et al., 2024). Together, these studies highlight the practical power of DIA phosphoproteomics in precisely characterizing the architectural dynamics of early plant stress-responsive networks.

Aside from phosphorylation, glycosylation has also been largely quantified by DIA-MS (Anjo et al., 2017). For instance, SWATH-MS was enabled to automate measurement of site-specific occupancy at many glycosylation sites through combining enzymatic deglycosylation and protease digestion (Xu et al., 2015). Additionally, a study presented an initial exploration of DIA-based quantification of N-linked intact glycopeptides, offering an alternative approach for quantitatively characterizing N-linked intact glycopeptides on a proteomic scale using Spectronaut. This method provides a means to investigate diseases associated with aberrant glycosylation (Dong et al., 2021). As glycosylation typically give rise to oxonium ions (from fragmentation of HexNAc), this feature was exploited in a DIA method termed OxoScan-MS where oxonium ion-containing scanning quadrupole profiles are extracted (White et al., 2024).

In plants, DIA-MS has been applied to a more limited extent for PTM analysis, yet it offers distinct advantages in low-input plant samples where comprehensive coverage is critical. Another study, which analyzed the dynamic pattern of protein carbonylation in rice embryos during germination (Zhang et al., 2016), enhances our understanding of the biochemical mechanisms involved in this process. A recent method CysQuant used DIA-MS to profile the degree of cysteine oxidation and the protein abundance simultaneously within samples (Huang et al., 2023). By nonisobaric, differential labelling with light and heavy iodoacetamide of reduced and oxidized cysteines, oxidation levels are quantified in a labeled-manner using plexDIA (Derks et al., 2023), while non-cysteine peptides inform on protein abundance by label-free quantitation (Huang et al., 2023). Such workflows illustrate how DIA-MS’s high reproducibility and multiplexing capabilities make it particularly well-suited for PTM studies in both animal and plant systems.

## Concluding remarks and future perspectives

DIA-MS has emerged as a powerful technology that seamlessly integrates deep proteome coverage with quantitative precision and reliability in plant proteomics. Recent developments in this field have moved beyond the simple detection of proteins, peptides, or PTMs in a limited number of samples. They now enable robust and reproducible quantification across medium to large scale plant studies involving diverse tissues, developmental stages, genotypes, treatments, and environmental conditions. This capacity is particularly valuable for plant stress biology, crop trait analysis, developmental regulation, and systems-level studies, where consistent and comprehensive proteomic datasets are required to reveal biologically meaningful regulatory mechanisms. Consequently, future DIA-MS-based plant proteomics should continue to emphasize data repeatability, proteome depth, quantitative accuracy, and cross-sample comparability. In addition, the application of DIA-MS to emerging areas such as plant SCP may provide new opportunities to resolve cell-type-specific proteome dynamics.

DIA-MS is a high-throughput technique enabling quantitative proteomic analysis. It has become increasingly useful for comparing protein abundance across diverse plant samples. Because plant tissues contain rigid cell walls, abundant pigments, phenolic compounds, carbohydrates, secondary metabolites, and highly abundant photosynthetic proteins, sample preparation remains a critical challenge for plant DIA-MS analysis. Optimized extraction, digestion, cleanup, and simplified sample preparation workflows are therefore essential for improving proteome coverage and quantitative reproducibility. In addition, the choice of DIA data analysis software and protein identification algorithms can influence the final protein identification and quantification results. For plant DIA-MS studies, the combined use or careful comparison of different software tools may help improve identification confidence, increase proteome coverage, and ensure robust quantitative outputs.

While traditional single omics approaches fail to explain the complete life activities of plants effectively, the integration of DIA-MS-based proteomics with transcriptomics, genomics, metabolomics, and other omics approaches offers a more comprehensive strategy for understanding plant regulatory networks. The convergence between proteomics and several other approaches offers new perspectives and enhanced understanding for these investigations. By integrating different omics techniques and simultaneous analysis of these datasets, transcriptional regulation processes as well as modifications and metabolic changes occurring within biological systems from multiple angles become possible. For example, DIA-MS data can be integrated with metabolomic datasets to identify enzymes associated with specific metabolic pathways, while changes in secondary metabolite abundance can help reflect plant physiological status under developmental, stress, or immune-response conditions. DIA-MS-based proteomics can also be combined with transcriptomics and genomics to support proteogenomic analysis, facilitating the discovery of novel peptides and the refinement of gene models and protein annotations (Song et al., 2023). This multidimensional omics approach offers a solution to the limitations of single omics approaches in explaining complex biological phenomena. Overall, the integration of DIA-MS with multi-omics approaches will provide a deeper and more systematic understanding of molecular regulation in plants.

We believe that continued development and application of DIA-MS in plant proteomics will facilitate the discovery of novel protein regulators and pathways, providing valuable insights for plant biology, crop improvement, and agricultural biotechnology. Emerging DIA approaches, such as 4D proteomics or single cell proteomics, are expected to further expand the depth and resolution of plant proteome research. With continued optimization of plant-compatible sample preparation, acquisition strategies, and computational workflows, these approaches may enable more precise identification of low-abundance regulatory proteins, membrane proteins, cell wall proteins, and components of complex signaling pathways. We firmly believe that leveraging the power of this proteomic methodology will facilitate the discovery of new and novel protein regulators and pathways, offering valuable insights and innovative ideas for biotechnological research in plants.
